# Effect of Human Amnion Epithelial Cells on the Acute Inflammatory Response in Fetal Sheep

**DOI:** 10.3389/fphys.2017.00871

**Published:** 2017-11-06

**Authors:** Alana Westover, Jacqueline M. Melville, Courtney McDonald, Rebecca Lim, Graham Jenkin, Euan M. Wallace, Timothy J. Moss

**Affiliations:** ^1^The Ritchie Centre, Hudson Institute of Medical Research, Clayton, VIC, Australia; ^2^Department of Obstetrics and Gynaecology, School of Clinical Sciences, Monash University, Melbourne, VIC, Australia

**Keywords:** hAECs, inflammation, fetus, lung, placenta, liver

## Abstract

Intra-amniotic (IA) lipopolysaccharide (LPS) injection in sheep induces inflammation in the fetus. Human amnion epithelial cells (hAECs) moderate the effect of IA LPS on fetal development, but their influence on the acute inflammatory response to IA LPS is unknown. We aimed to determine the effects of hAECs on the acute fetal inflammatory response to IA LPS. After surgical instrumentation at 116 days' gestation (d) ewes were randomized to 1 of 4 groups at 123 d: IA LPS (10 mg) and intravenous (IV) saline (*n* = 8), IA LPS and IV hAECs (*n* = 6), IA saline and IV saline (*n* = 5) or IA saline and IV hAECs (*n* = 5). IV injections were administered immediately after IA injections. Serial fetal blood samples were collected. At 125 d, placental, fetal lung and liver samples were collected. IA LPS increased inflammatory cell recruitment in the placenta and lungs, increased IL-1β and IL-8 mRNA levels in the lungs and increased serum amyloid A3 (SAA3) and C-reactive protein (CRP) mRNA levels in the liver. IV hAECs reduced fetal lung inflammatory cell recruitment but did not otherwise alter indices of placental, fetal lung or liver inflammation. The acute fetal inflammatory response to IA LPS is not substantially altered by IV hAEC treatment.

## Introduction

Chorioamnionitis, inflammation of the chorion and amnion, affects approximately 5% of all human pregnancies (Tita and Andrews, 2010), but its incidence is much higher in preterm births, where it is likely the principal causative factor (Goldenberg et al., 2008). Chorioamnionitis is present in approximately 75% of preterm births occurring before 24 weeks' gestation (Lahra et al., 2009). A consequence of intrauterine inflammation is altered fetal development and risk of morbidity and mortality in preterm infants (Hofer et al., 2013).

Chorioamnionitis can be modeled in sheep by intra-amniotic (IA) injection of lipopolysaccharide (LPS) (Kramer et al., 2010). Experiments using this sheep model of chorioamnionitis have revealed multiple effects on fetal development and perinatal physiology consistent with observations in humans (Galinsky et al., 2013b). IA LPS injection elicits a fetal inflammatory response within hours of injection that is maximal at approximately 2 days after injection (Kramer et al., 2001). The fetal pulmonary consequences of IA LPS injection include changes in lung structure and increased surfactant production that are evident 1 week after LPS injection (Jobe et al., 2000; Westover et al., 2012); these changes likely explain the reduced risk of respiratory distress syndrome in human infants born after exposure to chorioamnionitis (Jobe, 2012).

The effects of IA LPS injection on lung developmental in sheep can be altered by human amnion epithelial cells (hAECs). Administration of hAECs to fetal sheep at the time of (and at 6 and 12 h after) IA LPS injection attenuated effects on lung development assessed 7 days later (Vosdoganes et al., 2011). Modulation of effects on lung development was accompanied by reduced levels of proinflammatory cytokines in the lungs, despite greater infiltration of inflammatory cells by 7 days after IA LPS (Vosdoganes et al., 2011). An anti-inflammatory effect of hAECs is the likely mechanism of action of these cells, as revealed by investigations *in vivo* (Murphy et al., 2011) and *in vitro* (McDonald et al., 2015). However, it is unknown whether the effect of hAECs on the fetal lung response observed at 7 days after IA LPS was due to modulation of the acute inflammatory response to IA LPS, or due to later modulation of lung inflammation.

We aimed to compare the acute inflammatory responses to IA LPS between fetal sheep that received either concomitant intravenous hAECs or vehicle. We hypothesized that administration of hAECs would decrease the initial inflammatory response to IA LPS in fetal sheep lungs.

## Methods

### Animal experimentation

The relevant Monash University Animal Ethics Committee approved all animal experimental procedures.

Pregnant ewes bearing single fetuses underwent aseptic surgery on day 116 (term is ~147 days) of gestation. Anesthesia was induced by maternal intravenous (IV) injection of sodium thiopentone (1 g, via jugular venepuncture) and, after intubation with a cuffed endotracheal tube, was maintained by inhalation of isoflurane (2–3% in oxygen) delivered by positive pressure ventilation. An incision was made in the midline of the ewe's abdomen, the uterus was palpated to maneuver the fetal head beneath the maternal incision, and a smaller incision was made through the uterine wall and fetal membranes. The fetal head was exteriorized and bespoke catheters made from polyvinyl tubing (Dural Plastics, Australia) and blunt needles (Terumo, USA) were inserted into a fetal carotid artery, jugular vein and the amniotic cavity. Fetal and amniotic catheters were exteriorized through a small incision in the ewe's flank and secured in plastic bags, which were placed under elasticized netting fitted around the ewe's torso.

Human amnion epithelial cells were isolated from term placentae delivered by elective cesarean section from healthy volunteers. Women gave written, informed consent for the collection of their placentae, in accordance with approval from the human research ethics committee at Monash Health. Cell isolation was performed using methods described previously (Murphy et al., 2010). For IV treatments, hAECs from three donors were thawed and combined, washed, counted and assessed for viability, then resuspended at 30 × 10^6^ cells/ml in sterile saline for administration.

On day 123 of pregnancy, ewes were randomized to 1 of 4 groups: LPS (from *E. Coli* 055:B5; 10 mg in 2 ml physiological saline) or saline (2 ml) was injected into the amniotic cavity, and hAECs (90 × 10^6^ suspended in 3 ml saline) or 3 ml of saline was injected via the fetal jugular vein catheter, immediately after the IA injection. There were 8 fetuses that received IA LPS and IV saline; 6 received IA LPS and IV hAECs; 5 fetuses received IA and IV saline; and 5 received IA saline and IV hAECs.

Samples of fetal arterial blood (3–5 ml) were collected immediately prior to IA and IV injections, and at 5, 10, 24, and 48 h after injection, for measurement of fetal blood gases and metabolites (Radiometer ABL 500 blood-gas analyser; Radiometer, Copenhagen, Denmark) and collection of plasma.

Forty-eight hours after IA and fetal IV injections, ewes and fetuses were killed (pentobarbitone overdose: 5 g via the maternal jugular vein catheter) for tissue collection. Fetal body weight and weights of major organs were recorded. Samples of liver and the lower lobe of the right lung were frozen in liquid N_2_. The upper lobe of the right lung was fixed at 30 cm H_2_O by instillation of 10% paraformaldehyde solution. A sample of chorioamnion was fixed by immersion in 10% paraformaldehyde solution.

### Enzyme linked immunosorbent assays

Plasma levels of IL-6, and IL-10 were measured by enzyme-linked immunosorbent assay (ELISA). Briefly, 96-well plates (Nunc Maxisorp) were pre-coated with monoclonal antibodies (Table 1) at a concentration of 2.5 μg/ml and incubated overnight at 4°C. After washing the plate with washing buffer (PBS, 0.05% Tween-20), plates were blocked for 1 h with PBS/0.1% BSA. Recombinant sheep proteins were used as standards. All standards and samples were serially diluted, loaded to wells (100 μl) in duplicate and incubated for 1 h at room temperature. Plates were washed five times between steps. Rabbit polyclonal anti-ovine antibodies (IL-6, diluted 1:5000; Serotec MCA1659) or mouse anti-bovine biotinylated antibody (IL-10, diluted 1:1000; Serotec MCA2111B) were added to wells and incubated for 1 h at room temperature. Detection was accomplished by incubating the conjugated enzyme for 1 h at room temperature and then the TMB substrate solution (tetramethylbenzidine, Thermo Fisher) for 20 min at room temperature. The color development reaction was stopped with 50 μl of 2M sulfuric acid. Plates were read on a micro-plate reader (SpectraMax i3) at 450 nm.

**Table 1:** ELISA details.

**Analyte**	**Monoclonal antibody**	**Dilution**	**Conjugated enzyme**	**Dilution**
IL-6	Recombinant ovine IL-6 (Serotec, MCA1659)	0.1 μg/ml	Swine anti-rabbit Ig-HRP conjugate	1:1000
IL-10	Mouse anti-bovine IL-10 (Serotec, MCA2110)	1:200	Streptavidin-HRP (Dako, P0397)	1:1000

### Immunohistochemistry

Fixed lung tissue and chorioamnion sections were processed for immunohistochemical staining of leukocytes using a CD45 antibody (diluted 1:100, MCA2220PE; AbD Serotec) (Westover et al., 2012). CD45-positive cells were counted in 3 random non-overlapping fields of view, by an operator blinded to treatment, using the Image-Pro Plus (Media Cybernetics) software program. The mean number of cells/field of view was calculated for each subject.

### Real-time PCR

Total RNA was extracted from fetal liver and lung samples using an extraction kit (RNeasy maxi (lung) or midi (liver) kit; Qiagen) according to the manufacturer's instructions. Reverse transcription was performed on RNA with the Superscript III First Strand synthesis system for real-time PCR kit as specified by the manufacturer (18080-051; Invitrogen).

Quantitative real-time PCR was used to measure mRNA levels for interleukin (IL)-1ß, IL-6, and IL-8 in lung tissue, and serum amyloid A3 (SAA3), C-reactive protein (CRP) and hepcidin in liver using primer sequences and reaction conditions described previously (Wallace et al., 2009; Polglase et al., 2014). The mRNA levels for each fetus were compared to the housekeeping gene, *18S*, and were calculated using the ΔΔCt method and are expressed relative to the mean of the IA saline + IV saline group.

### Data analysis

Data analysis was performed using Prism (6.0 g, Graphpad Software). Data are presented as mean ± SEM. Variables measured at a single time point were compared between groups using 2-way analysis of variance (ANOVA) with IA (LPS or saline) and IV (hAECs or saline) injections as factors. Serial data were compared between groups using repeated measures ANOVA with IA and fetal IV treatments and time as factors (between or within, respectively). *Post-hoc* tests to identify differences between groups were performed using SPSS Statistics 22 (IBM).

## Results

### Fetal blood gases

**Figure 1:**
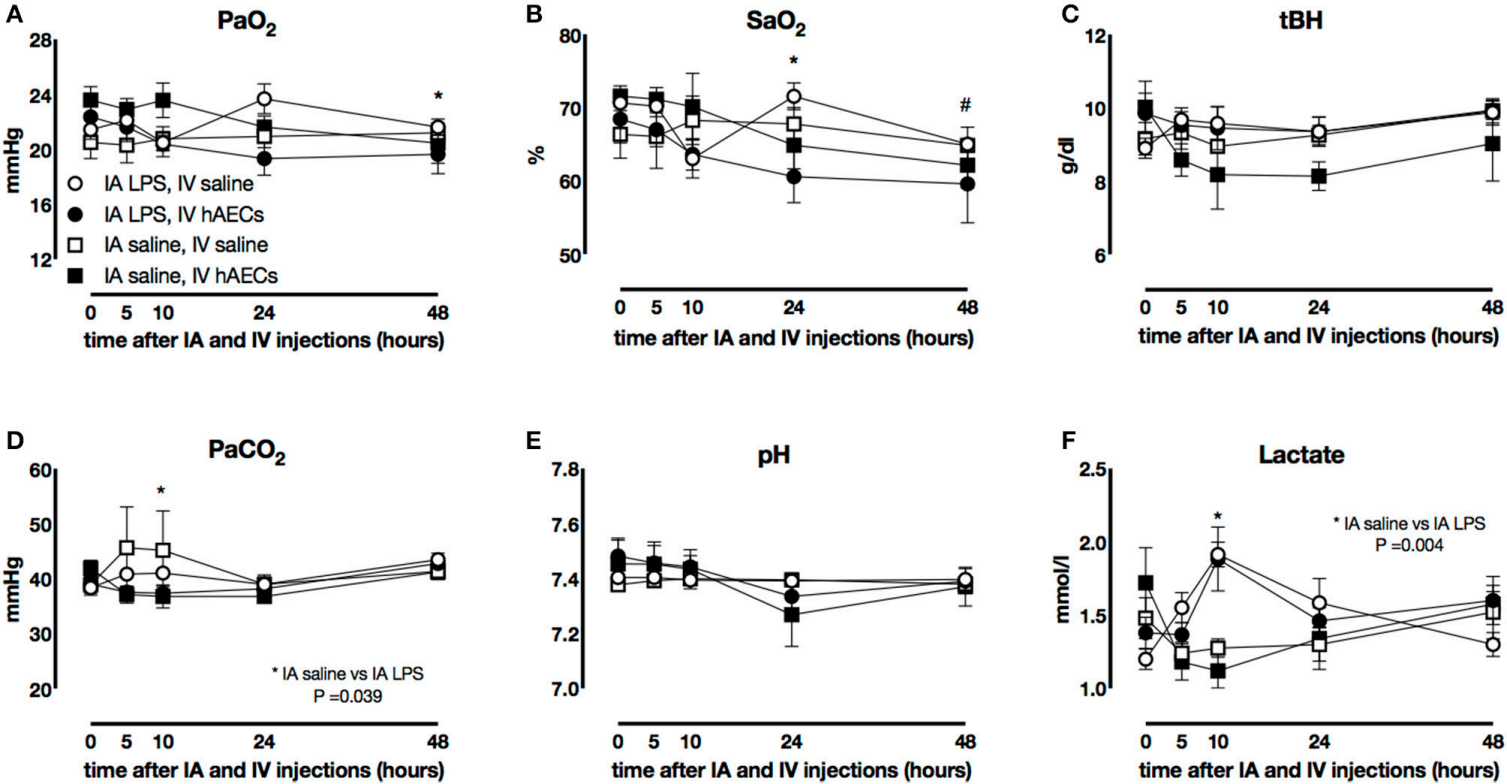
Fetal blood gases. Fetal PaO_2_ **(A)** decreased over time in groups that received IV hAECs (^*^P = 0.019), as did SaO_2_ (**B**: ^*^P = 0.04; ^#^*P* < 0.005). Total hemoglobin concentrations **(C)** were not significantly different between groups. Fetal PaCO_2_ **(D)** demonstrated a transient increase in groups that received IA LPS compared to IA saline and a significant interaction between time and IV hAECs. Fetal pH **(E)** was not significantly different between groups. Fetal blood **(F)** lactate concentrations demonstrated a transient increase in groups that received IA LPS injections. Data are expressed as mean ± SEM.

Fetal PaO_2_ (Figure 1A) was lower 48 h after IV hAECs than before IV injection (*P* = 0.019) but there was no effect of IA (LPS or saline) injection. Fetal SaO_2_ (Figure 1B) was also lower after IV hAECs (at 24 and 48 h) than before IV injection (*P* = 0.04 and *P* < 0.005, respectively) but there was no difference between IA LPS or saline groups. Fetal PaCO_2_ (Figure 1D) 10 h after IA LPS was higher than in fetuses that received IV saline (*P* = 0.039). Fetal blood lactate (Figure 1F) concentrations were higher 10 h after LPS administration (*P* = 0.004). Fetal pH (Figure 1E) and hemoglobin (Figure 1C) were not different between groups.

### Fetal organ weights

**Figure 2:**
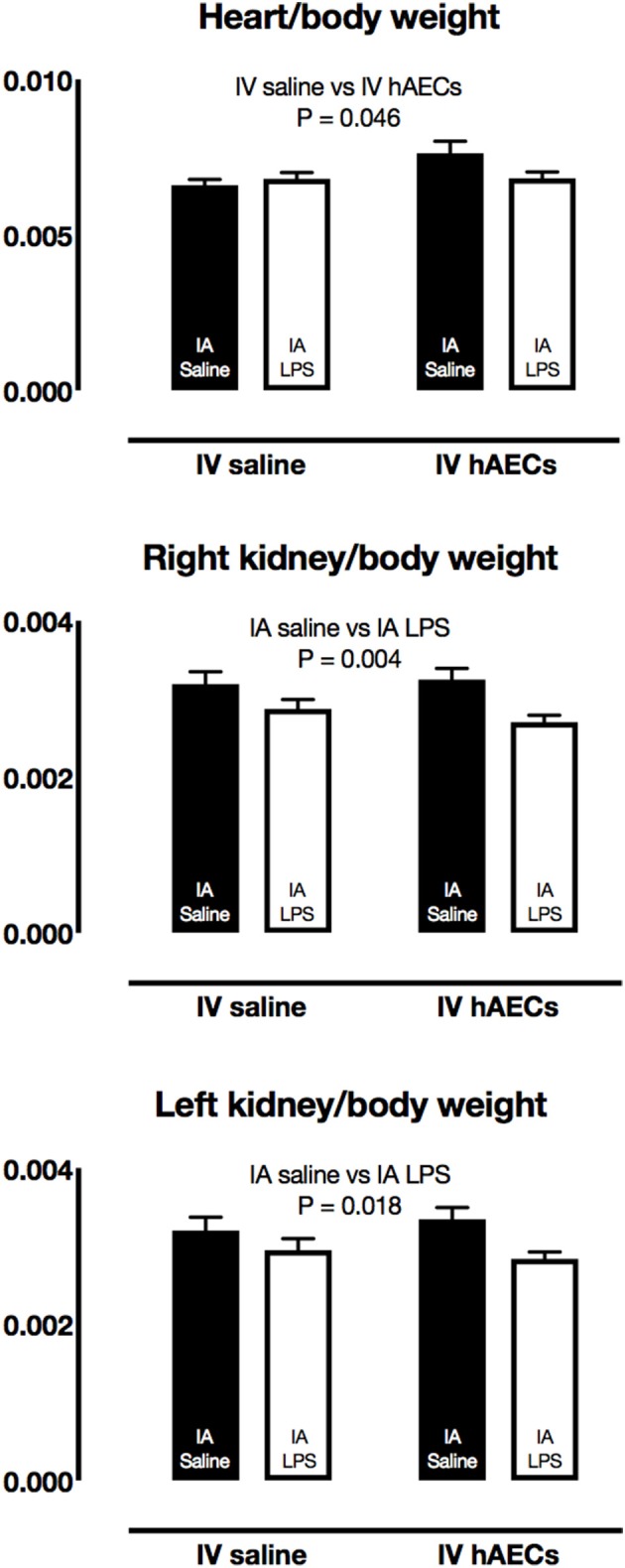
Fetal organ/body weight ratios. Fetal heart/body weight ratios were higher in IV hAECs groups than in IV saline groups (**A**: *P* = 0.046), whereas right **(B)** and left **(C)** kidney/body weight ratios were lower in groups that received IA LPS than IA saline groups (*P* = 0.004 and 0.018, respectively). Data are expressed as mean ± SEM.

There were no differences in fetal body weights, or in brain, lung, liver, spleen, thymus or adrenal/body weight ratios between groups (Table 2). Heart/body weight ratios were higher (Figure 2A; *P* = 0.04) in fetuses that received a combined treatment of IA saline and IV hAECs, compared to other groups. Left (Figure 2C; *P* = 0.02) and right (Figure 2B; *P* = 0.003) kidney/body weight ratios were lower in fetuses exposed to IA LPS than in IA saline groups.

**Table 2:** Fetal body weights and relative organ weights.

	**IA saline + IV saline**	**IA saline + IV hAECs**	**IA LPS + IV saline**	**IA LPS + IV hAECs**
Body weight (kg)	3.64 ± 0.59	3.89 ± 0.57	4.09 ± 0.39	4.12 ± 0.52
Brain (g kg^−1^)	12.48 ± 2.07	11.38 ± 1.76	11.23 ± 1.04	11.40 ± 1.47
Heart (g kg^−1^)	6.60 ± 0.44	*7.62 ± 0.87*	6.81 ± 0.57	6.83 ± 0.49
		***P** = **0.04***		
Liver (g kg^−1^)	36.72 ± 2.84	34.40 ± 4.76	35.53 ± 4.60	32.56 ± 2.69
Left lung (g kg^−1^)	13.63 ± 2.56	13.67 ± 1.12	13.78 ± 1.57	13.97 ± 2.05
Right lung (g kg^−1^)	19.78 ± 3.46	19.92 ± 1.96	20.55 ± 2.25	21.46 ± 2.97
Left kidney (g kg^−1^)	3.20 ± 0.42	3.34 ± 0.34	*2.95 ± 0.41*	*2.84 ± 0.22*
			***P*** = **0.02**	
Right kidney (g kg^−1^)	3.18 ± 0.39	3.25 ± 0.32	*2.87 ± 0.34*	*2.70 ± 0.21*
			***P*** = **0.003**	
Left adrenal (g kg^−1^)	0.054 ± 0.0059	0.049 ± 0.0073	0.057 ± 0.0095	0.058 ± 0.0074
Right adrenal (g kg^−1^)	0.055 ± 0.0048	0.049 ± 0.0098	0.058 ± 0.0010	0.053 ± 0.0061
Spleen (g kg^−1^)	1.89 ± 0.30	1.73 ± 0.37	2.03 ± 0.18	1.77 ± 0.28
Thymus (g kg^−1^)	4.59 ± 0.94	4.64 ± 0.41	4.42 ± 0.12	4.46 ± 0.67

*Values are mean ± SEM. Bold and italic values indicate values that are statistically significant*.

### Systemic inflammation

**Figure 3:**
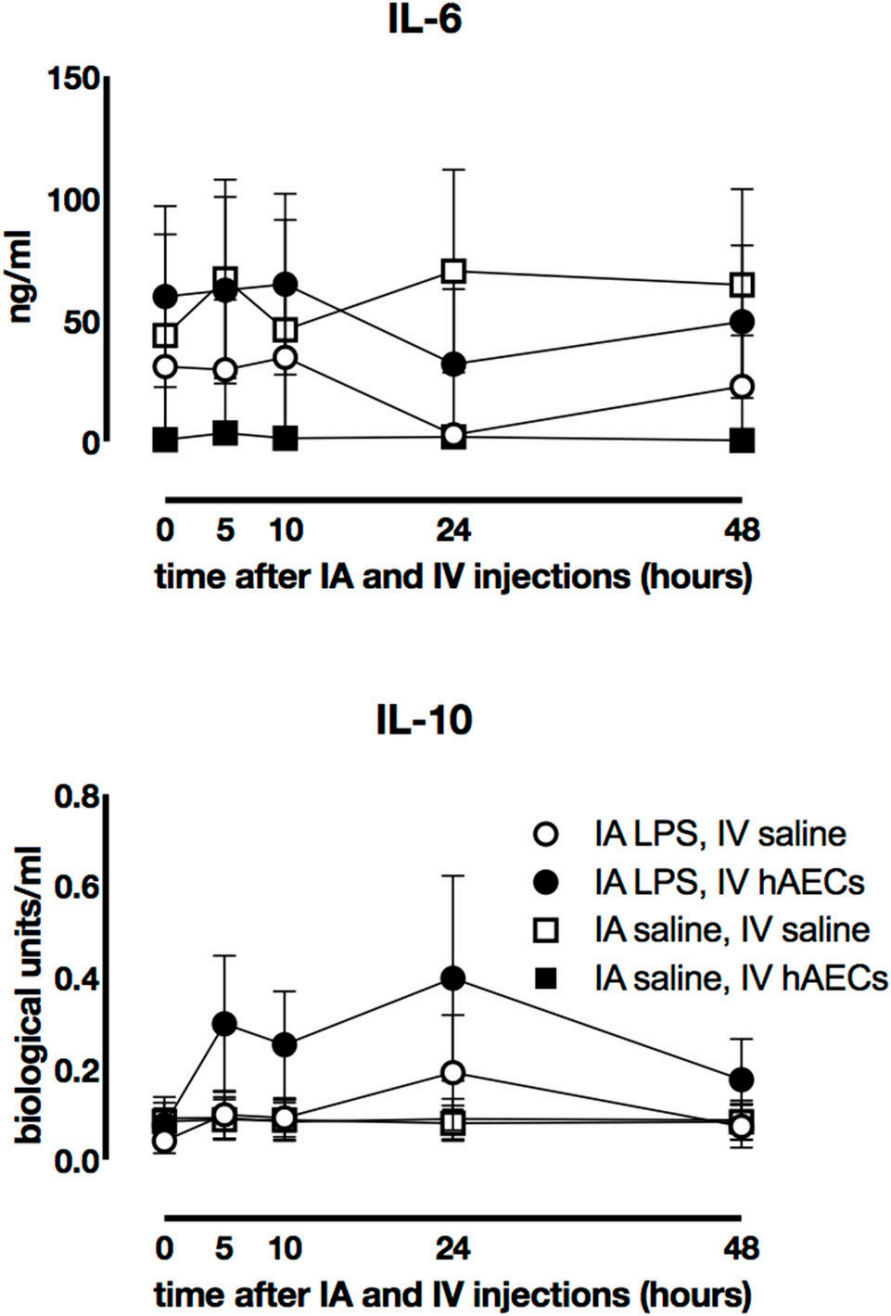
Effect of IA LPS and IV hAECs on systemic cytokine expression. Fetal plasma IL-6 **(A)** and IL-10 **(B)** concentrations were not significantly different between groups. Data are expressed as mean ± SEM.

Fetal plasma IL-6 (Figure 3A) and IL-10 (Figure 3B) concentrations were not significantly different between IA LPS and IV hAECs treatment groups.

### Inflammation in the chorioamnion

**Figure 4:**
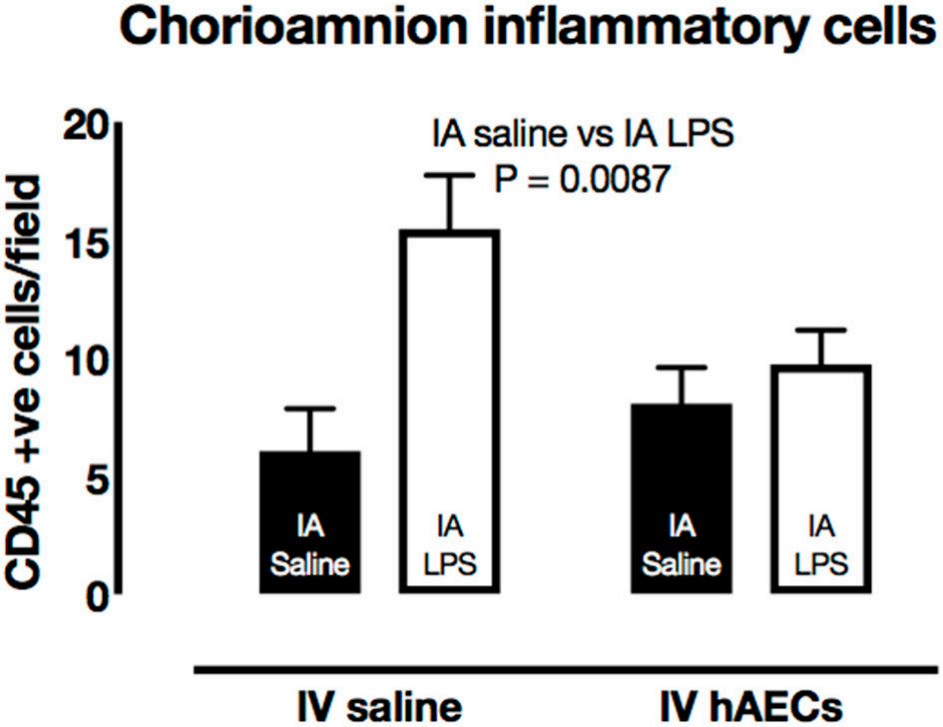
Effect of IA LPS and IV hAECs on expression of inflammatory cells in the chorioamnion. The number of CD45-positive cells per field of view was higher in chorioamnion from groups given IA LPS compared to groups that received IA saline. Data are expressed as mean ± SEM.

The number of CD45-positive cells in the chorioamnion was higher (Figure 4; *P* = 0.009) in groups that received IA LPS relative to those that received IA saline. There was a strong trend toward an interaction between IA and IV treatments (*P* = 0.055); *post-hoc* comparisons showed higher CD45-positive cell numbers for the group that received IA LPS and IV saline (*P* = 0.008).

### Inflammation in the lung

**Figure 5:**
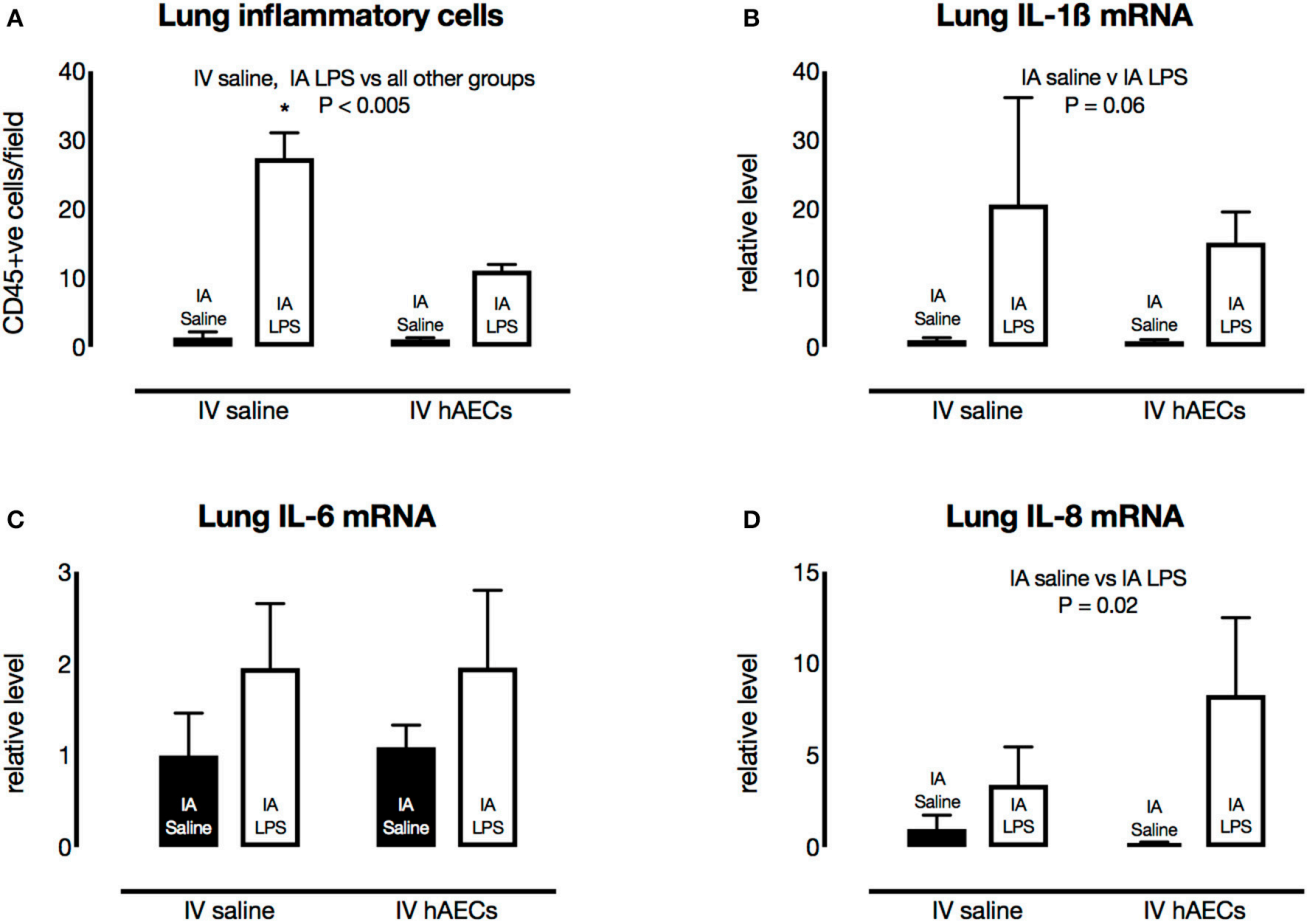
Effect of IA LPS and IV hAECs on expression of inflammatory markers in the fetal lungs. **(A)** The number of CD45-positive cells per field of view was significantly higher after IV saline and IA LPS than other groups (^*^P < 0.005). Fetal lung mRNA levels of IL-1β **(B)** and IL-6 **(C)** were not significantly altered by IA LPS or IV hAECs. Fetal lung IL-8 mRNA levels **(D)** were higher after IA LPS than after IA saline (*P* = 0.002). Data are expressed as mean ± SEM, relative to the mean of the IV saline, IA saline group.

The number of CD45-positive cells in the lungs was higher (Figure 5A; *P* = 0.003) in groups that received IA LPS compared to those that received IA saline. However, there were fewer (*P* < 0.0001) inflammatory cells in the IA LPS group that received IV hAECs compared to the LPS group that received IV saline.

Fetal lung IL-1β (Figure 5B) and IL-6 (Figure 5C) mRNA levels tended higher than control levels in groups that received IA LPS, although these increases were not statistically significant. Fetal lung IL-8 mRNA levels were higher (Figure 5D; *P* = 0.02) in IA LPS groups relative to IA saline groups. Cytokine mRNA responses were not significantly different between IV treatments.

### Inflammation in the liver

**Figure 6:**
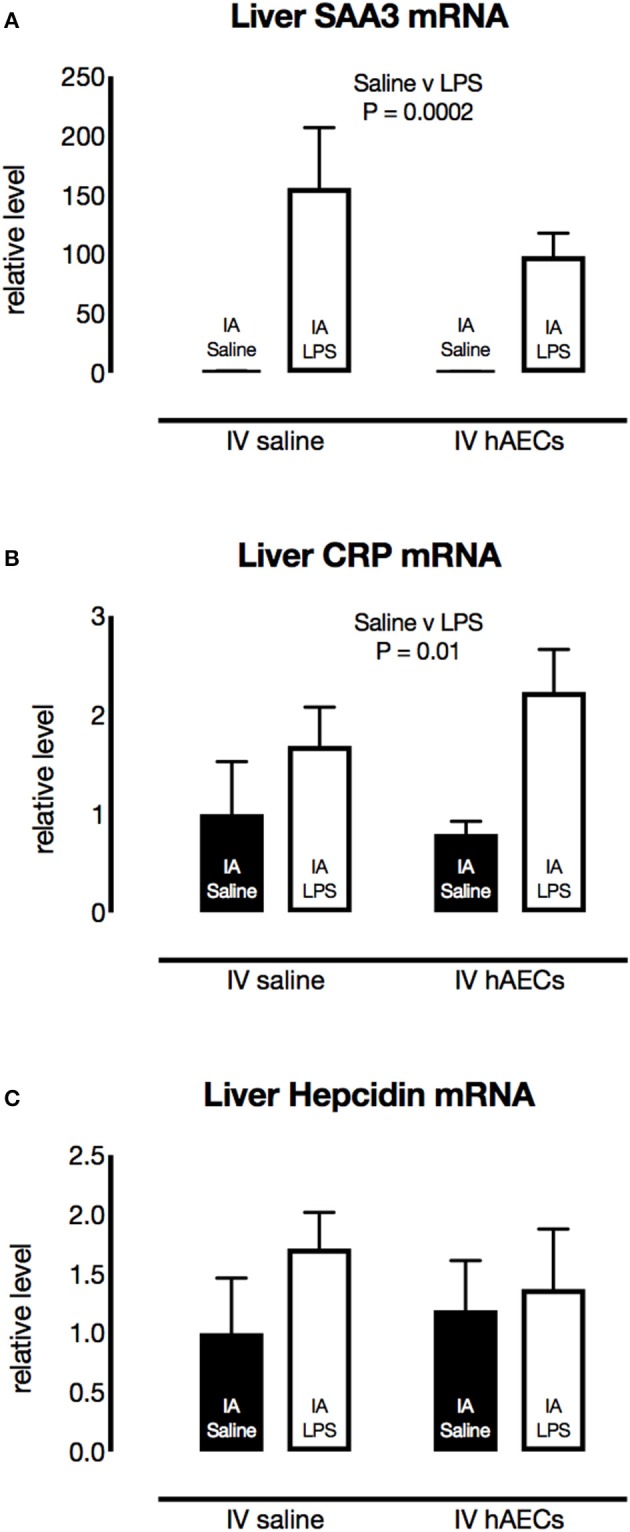
Effect of IA LPS and IV hAECs on expression of inflammatory markers in the fetal liver. Fetal liver mRNA levels of SAA3 **(A)** and CRP **(B)** were higher in groups that received IA LPS than in IA saline groups (*P* = 0.002 and 0.01, respectively). Hepcidin mRNA levels **(C)** were not altered by IA LPS or IV hAECs. Data are expressed as mean ± SEM, relative to the mean of the IV saline, IA saline group.

Fetal liver SAA3 mRNA levels were over 100-fold (Figure 6A; *P* = 0.0002) higher in groups that received IA LPS compared to IA saline. CRP mRNA levels (Figure 6B; *P* = 0.01) in the fetal liver were also higher in groups that received IA LPS. Hepcidin mRNA levels (Figure 6C) in fetal liver tissue were not altered by IA LPS. There was no difference in liver SAA3, CRP or Hepcidin between IV Saline or hAEC groups.

## Discussion

Intravenous administration of hAECs to fetal sheep reduced the initial inflammatory cell infiltration of the fetal lungs in response to IA LPS injection, consistent with a capacity of the cells to modulate the acute inflammatory response. However, increases in pro-inflammatory cytokine gene expression in the fetal lungs, and indices of systemic inflammation, were not altered by intravenous hAEC administration. These observations suggest that previously observed longer-term changes in fetal inflammation and effects on development in sheep (Vosdoganes et al., 2011; Yawno et al., 2013) result from modulation of the progressive effects of fetal inflammation and injury, not modulation of the initial acute inflammatory response.

Our previous studies show clearly that hAECs, administered by systemic and/or respiratory routes can modulate lung inflammation, and consequent effects on lung development and remodeling, when administered during or after establishment of numerous injurious stimuli including bleomycin, hyperoxia, LPS and mechanical ventilation (Murphy et al., 2011; Vosdoganes et al., 2011, 2013; Hodges et al., 2012). The results from our current study suggest the benefit of hAEC therapy does not lie in the ability of the cells to attenuate the initial phase of inflammation. This supports the concept that hAECs act by modulating the action of immune cells during the propagation of inflammation (McDonald et al., 2015).

IA LPS caused inflammation equivalent to that which occurs with chorioamnionitis in our current study, as indicated by inflammatory cell infiltration into the chorioamnion. IA LPS-induced chorioamnionitis is associated with a low-grade fetal inflammatory response (Kramer et al., 2001; Nitsos et al., 2002), as demonstrated by our observation of elevated SAA3 and CRP mRNA levels in the fetal liver, consistent with previous observations (Kemp et al., 2013). Despite evidence of a systemic inflammatory response to IA LPS, we did not observe effects of either IA LPS or IV hAECs on fetal plasma IL-6 concentrations. This observation replicates a previous report that circulating concentrations of this pro-inflammatory cytokine were not significantly elevated in chronically instrumented fetal sheep during the 2 days after IA LPS (Grigsby et al., 2003). Our study extends this observation to show that fetal plasma IL-6 concentrations are not altered by IV hAECs.

Fetal plasma IL-10 concentrations were not significantly altered by LPS or hAECs but there was a tendency for higher IL-10 levels in fetuses after IA LPS and IV hAECs. Previous reports of the effects of hAECs in the livers of mice with hepatic fibrosis *in vivo* (Manuelpillai et al., 2010) and *in vitro* (Magatti et al., 2015) show an elevation in levels of this anti-inflammatory cytokine, which has a role in programming an M2-like anti-inflammatory phenotype in macrophages. This effect is consistent with M2-like polarization of macrophages by hAECs and hAEC-conditioned media (Tan et al., 2014), a process that is dependent on T regulatory lymphocytes (Tan et al., 2015).

The effect of IA LPS on fetal lung inflammation is well described (Kallapur et al., 2001; Kramer et al., 2001; Moss et al., 2002a; Westover et al., 2012). LPS-induced inflammatory cell infiltration was attenuated by IV hAECs in our experiment, consistent with the observation of reduced numbers of macrophages and neutrophils in bleomycin-treated mice 6 days after IP injection of hAECs (Murphy et al., 2011): however, in our experiment, inflammatory cell recruitment was not reduced to control levels at the time of examination (4 days after treatment). Our results contrast with our earlier report of increased inflammatory cell infiltration in the lungs by 7 days after IV and intratracheal administration of hAECs following IA LPS (Vosdoganes et al., 2011). Thus, there may be temporal differences in the effects of hAECs on fetal lung inflammatory cell recruitment after IA LPS. Alternatively, the elevation in lung inflammatory cell numbers observed by us previously may be a consequence of the route of (tracheal) administration of hAECs. Increases in IL-6 mRNA in the fetal lungs have been observed at 24 and 48 h after IA LPS injection (Kallapur et al., 2001), and we observed a similar effect in this study, although the effect did not attain statistical significance. Intravenous administration of hAECs did not alter mRNA levels for proinflammatory cytokines in the lungs of fetal sheep in our current study, in contrast to the effects on cytokine levels observed 7 days after IA LPS and fetal IV hAEC administration (Vosdoganes et al., 2011). These contrasting findings likely reflect temporal changes in both the fetal response to IA LPS and to IV hAECs.

A possible limitation of our study, as it relates to our previous experiments in fetal sheep, is that hAECs were administered only IV to the fetus, rather than by IV and intratracheal routes (Vosdoganes et al., 2011; Hodges et al., 2012). IV administration alone was chosen for this study because we were interested in the capacity of hAECs to alter the acute fetal systemic response to IA LPS. It is possible that administration of cells to the fetal lungs would have a different effect than IV treatment in this animal preparation. The lung is the likely primary site of fetal inflammation in response to IA LPS (Moss et al., 2002b; Kemp et al., 2013), so potential modulation of fetal lung inflammation by local administration of hAECs could alter subsequent indices of systemic inflammation. However, our previous observations suggest IV administration is equivalent to intra-tracheal and/or IV treatment in this respect (Vosdoganes et al., 2011). Little is known about the optimal route of cell therapy for respiratory applications that have been reasonably well studied, such as acute respiratory distress syndrome (McIntyre et al., 2016), let alone for modulation of perinatal inflammatory responses.

The acute effect of IA LPS on fetal PaCO_2_ and lactate concentrations observed in our experiment is consistent with previous studies (Westover et al., 2012; Galinsky et al., 2013a), and is likely attributable to transiently altered placental function or fetoplacental metabolism (Nitsos et al., 2002). The abolition of the inflammation-induced transient fetal hypercapnia by hAECs suggests inhibition of these underlying processes. This observation is consistent with the prevention of IA LPS-induced inflammatory cell recruitment in the chorioamnion by IV hAEC administration. The small effect of hAEC administration on fetal oxygenation that we observed was small and is inconsistent with our previous observations of the effects of hAECs in fetal and neonatal lambs. We consider the small magnitude of this response would be insufficient to have deleterious effects but it may indicate some unknown effect of hAECs on the fetus or placenta. Replication and further investigation are required.

IA LPS altered the weights of the fetal kidneys (reduced) and heart (increased), contrasting with the lack of effect on organ weights we observed previously in fetuses delivered 7 days after IA LPS. A reduction in nephron number was, however, observed in fetal sheep 7 days after IA LPS (Galinsky et al., 2011). Relative heart weight was increased in fetuses treated with IA saline and IV hAECs but the short time-scale within which we observed an apparent increase in heart weight suggests that it is not the consequence of cardiac hypotrophy. The only other cardiac effect of hAEC administration that has been reported is alteration the vasculature of the heart in mice (Roy et al., 2015).

In summary, our study demonstrates that IV hAECs do not substantially alter the acute inflammatory response to IA LPS in fetal lungs and liver. This observation suggests that the previously reported modulation of inflammation-induced changes in fetal lung development by hAECs results from regulation of the ongoing inflammatory response after its establishment. This is a positive characteristic of hAECs that offer promise as a therapy to reduce deleterious effects of inflammation in the context of perinatal medicine.

## Author contributions

Conception or design of the work (CM, RL, GJ, EW, and TM), acquisition, analysis, or interpretation of data for the work. (AW, JM, CM, RL, GJ, EW, and TM), drafting the work or revising it critically for important intellectual content (AW, JM, CM, RL, GJ, EW, and TM). All authors: Approved the final version of the manuscript (AW, JM, CM, RL, GJ, EW, and TM), agree to be accountable for all aspects of the work in ensuring that questions related to the accuracy or integrity of any part of the work are appropriately investigated and resolved (AW, JM, CM, RL, GJ, EW, and TM), all persons designated as authors qualify for authorship, and all those who qualify for authorship are listed (AW, JM, CM, RL, GJ, EW, and TM).

### Conflict of interest statement

The authors declare that the research was conducted in the absence of any commercial or financial relationships that could be construed as a potential conflict of interest.
